# Noninvasive assessment of foot perfusion in cholesterol-fed rabbits using dynamic volume perfusion CT with an upslope method

**DOI:** 10.1038/s41598-022-12756-7

**Published:** 2022-05-25

**Authors:** Hooney Daniel Min, Saebeom Hur, Jae Hwan Lee, Chang Jin Yoon, Won Seok Choi, Seunghyun Lee

**Affiliations:** 1 Department of Radiology, Seoul National University Bundang Hospital, 82, Gumi-ro 173beon-gil, Bundang-gu, Seongnam-si, Gyeonggi-do 13620 South Korea; 2 Department of Radiology, Seoul National University College of Medicine, 101 Daehak-ro, Jongno-gu, Seoul, 03080 Republic of Korea; 3 Department of Radiology, Seoul National University Hospital, 101 Daehak-ro, Jongno-gu, Seoul, 03080 Republic of Korea

**Keywords:** Vascular diseases, Medical imaging

## Abstract

To evaluate the feasibility of dynamic foot volume CT with the upslope method and to demonstrate macrovascular reactivity and microvascular perfusion during cuff-induced reactive hyperemia state in cholesterol-fed rabbits. 30 New Zealand male rabbits were divided into 2 groups: dietary hypercholesterolemia (n = 10) and normal diet control (n = 20). To measure for macrovascular reactivity, perfusion parameters of the left posterior tibial artery was measured at baseline and at reactive hyperemia state. For the evaluation of microvascular perfusion, color-coded perfusion map of the plantar dermis was generated for perfusion CT scan by an in-house developed dedicated analysis software based on upslope method. Dermal perfusion values were measured and analyzed before and after cuff-induced reactive hyperemia. Foot dynamic volume CT with the upslope method demonstrated significant impairment of both macrovascular reactivity and microvascular perfusion in cholesterol-fed rabbits without significant macrovascular lesions during cuff-induced reactive hyperemia (CRH) state. Arterial time-to-peak of cholesterol-fed rabbits failed to show acceleration while chow-fed rabbits showed significant decrease in time. Microvascular perfusion calculated by perfusion value (P < 0.01) and perfusion ratio (*P* = .014) showed decreased microvascular perfusion in cholesterol-fed rabbits compared to chow-fed rabbits during CRH state. Post-CT pathologic examination revealed decreased endothelial cell density in cholesterol-fed rabbits (P < 0.001). Foot perfusion CT using upslope method provides perfusion parameters for large arteries and a perfusion map of the foot during cuff-induced reactive hyperemia in cholesterol-fed rabbits. It may be a useful tool to assess microvascular reactivity in patients with peripheral artery disease but no apparent macrovascular lesions.

## Introduction

Atherosclerotic peripheral arterial disease (PAD) is a global health problem, afflicting over 200 million people worldwide^1–3^. PAD is the third leading cause of atherosclerotic cardiovascular morbidity, following coronary artery disease and stroke^1^. Disability and mortality associated with PAD has significantly increased over the last 20 years^4^.

Ankle brachial index (ABI) is the most readily available, and validated method to screen and diagnose PAD^5^. ABI relies on macroscopic measurements of pressure gradient in the limbs to provide important diagnostic and prognostic information, such as risk of death, myocardial infarction and stroke^6,7^. Yet, despite the diagnostic and prognostic utility, ABI does not give any anatomical information. It is also not applicable to evaluate non-compressible arteries with heavy calcification or below-ankle diseases^8,9^. Other modalities such as peripheral artery tonometry (PAT) or flow mediated dilatation (FMD), while limited in use, can evaluate macrovascular reactivity but do not provide any anatomical information. Modalities that provide anatomic information for the evaluation of PAD such as nuclear imaging, computed tomographic (CT) angiography, magnetic resonance (MR) angiography, and conventional angiography visualize macrovascular lesions but do not evaluate microvascular perfusion^10–12^. The ideal test for PAD would provide both macrovascular and microvascular information as well as anatomical details to assess disease severity, guide decision-making, and predict treatment response. Therefore, a pragmatic modality to concurrently evaluate arterial blood flow, microvascular foot perfusion and anatomic lesions is imperative.

Perfusion imaging has been widely used to diagnose and treat coronary artery diseases and cerebral ischemic diseases^13–15^. In evaluating cerebral ischemic diseases, perfusion CT or MRI allows for the visualization of ischemic penumbra zones, providing practical information on brain tissue viability^13,14^. In the evaluation of coronary artery disease, the concept of “myocardial perfusion reserve” allows for the evaluation of the surrounding microvasculature’s ability to meet tissue demands upon stressors^16^. Perfusion reserve, defined as the ratio of global blood flow at stress vs. rest, allows for identification of at-risk tissue. Widely used in the clinic, stress testing with adenosine can induce vasodilatation and hyperemia which allows for the identification of myocardial tissue with hypoperfusion in asymptomatic individuals without abnormalities on conventional angiography^16^. These imaging modalities provide important information to non-invasively identify areas at risk and guide treatment. However, perfusion imaging modalities have yet to be applied for the evaluation of the foot for patients without critical limb ischemia.

In our previous study, we showed that foot perfusion CT with the upslope method can provide both qualitative and quantitative assessment of flow in the foot of patients with PAD^17^. In addition, we identified the presence of a dermal hyperperfusion band in the rabbit’s feet that served as the region of interest in evaluating foot tissue perfusion^17^.

Here, we evaluated the feasibility of dynamic volume CT with the upslope method during cuff-induced hyperemia, to demonstrate macrovascular reactivity and microvascular perfusion in cholesterol-fed rabbits without significant macrovascular lesions.

## Materials and methods

### Statement

All experiments and methods were performed in accordance with relevant guidelines and regulations. All experimental protocols were approved by a named institutional/licensing committee. Specifically, animal research protocol was approved by Institutional Animal Care and Use Committee of Seoul National University Bundang Hospital. The study was carried out in compliance with the ARRIVE guidelines. All animals were euthanized with an intravenous injection of a lethal amount (7–10 ml) of xylazine hydrochloride under deep anesthesia.

### Animal model preparation

**Figure 1:**
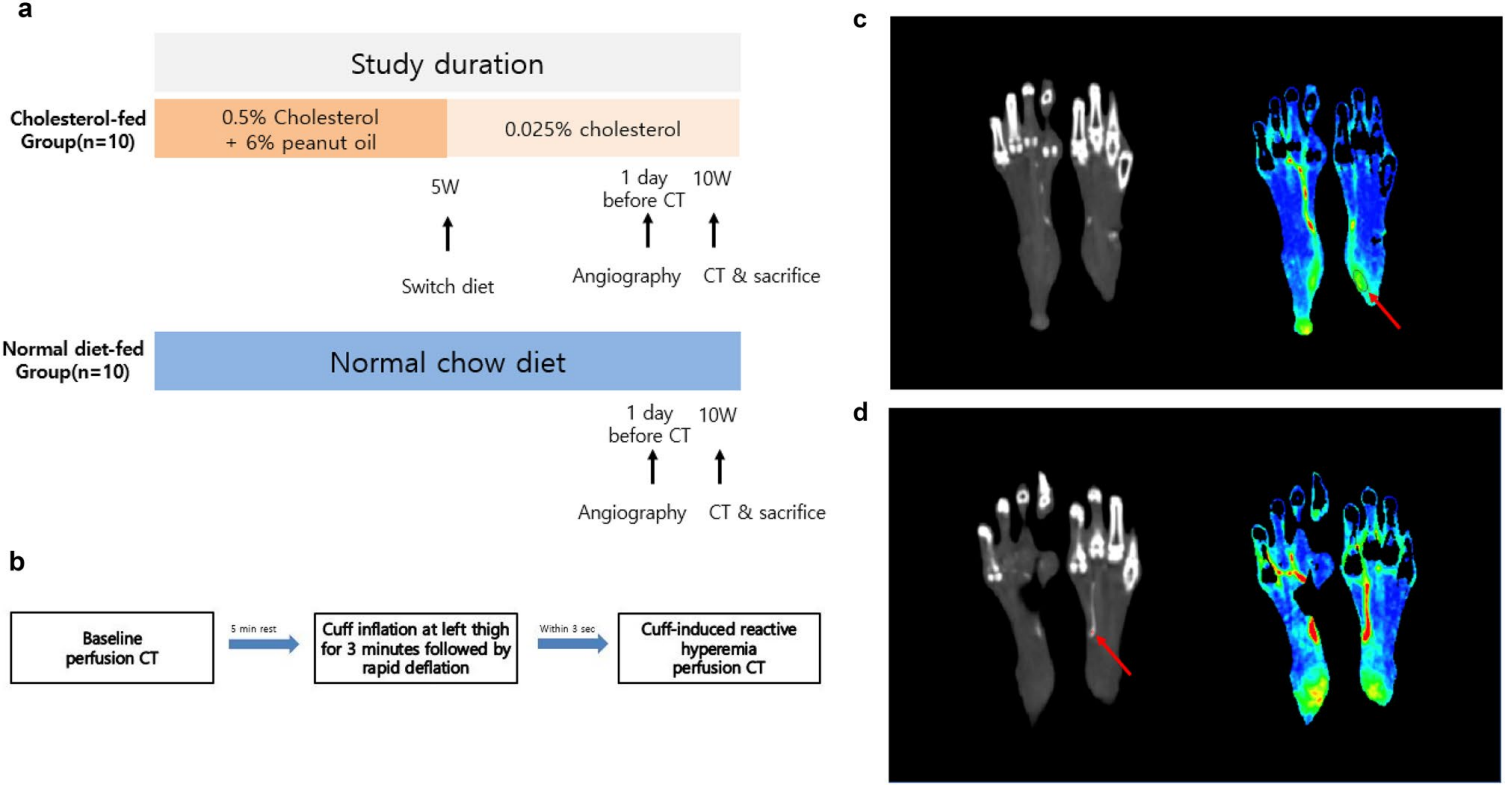
(**a**) Study protocol for cholesterol-fed rabbits. (**b**) Timeline of CT perfusion protocol. (**c**) Color-coded perfusion map reconstructed from perfusion CT. Microperfusion values were measured at selected region of interest (red arrow) in the hyperperfusion tissue in the rabbit’s heels on reconstructed image. (**d**) Color-coded perfusion map reconstructed from perfusion CT. CT value of tibial artery was measured on reconstructed CT image (red dot and red arrow).

Thirty New Zealand white male rabbits weighting 3000–3500 g were used in our study. All animals were housed in cages with a 12-h, light/dark cycle and ad libitum access to water. For anesthesia, 5 mg/kg body weight tiletamine-zolazepam (Zoletil 50; Virbac, Carros, France) and 2 mg/kg body weight of 2% xylazine hydrochloride (Rompun; Bayer, Seoul, Republic of Korea) were injected intramuscularly in the posterior thigh. Atherosclerotic diet was administered as described in previous studies^18–22^. The 30 rabbits were randomly divided into two groups by using random numbers were generated using the standard = RAND() function in Microsoft Excel. Other strategy to minimize potential confounders were not used. 10 rabbits in the cholesterol-fed group were given an atherogenic diet consisting of 0.5% cholesterol and 6% peanut oil for 5 weeks and then switched to a diet containing lower cholesterol (0.025%) for another 5 weeks to prevent liver failure^18,22^. The 20 rabbits in the control group were fed normal rabbit chow for all study periods. Pre-experimental exclusion criteria were set to exclude animals who may develop hepatic failure and poor general condition from cholesterol diet. No animals were excluded from the experiment. Random number generators were used for randomization Study protocol for induction of cholesterol-fed diet is schematically shown in Fig. 1.

### Sample size calculations

As no pilot data has been collected for these experiments, a standardized effect size was used for the power analysis. To detect a moderate to large clinically relevant effect size with a Cohen’s d of 1.4. A power analysis using the two-tailed student’s t-test, Sidak corrected for 1 comparison, with an alpha of 0.05 and a power of 0.8 was performed. From this analysis it was found that at least 10 animals in each group would be required.

### Angiography

All rabbits underwent angiography to evaluate the presence of significant stenosis or occlusion of bilateral aortoiliac and femorotibial arteries, one day prior to CT scan. After sedation, rabbits were placed in supine position and right central ear artery of each rabbit was cannulated using 16 gauze IV cannula. To access the infrarenal aorta, a 2.0-Fr microcatheter (progreat a, Terumo, Tokyo, Japan) was advanced via the ear artery to the iliofemoral artery and femorotibial angiography was performed.

### Foot perfusion CT

Perfusion CT protocol similar to previous experiment was performed to evaluate foot perfusion status of each rabbit as detailed in a previous study^17^. In brief, each animal was laid down on the table of a 64-detector CT scanner (Ingenuity; Philips Medical, Eindhoven, the Netherlands) with 4-cm z-axis coverage in volume scan mode. First, baseline perfusion CT scan was obtained by using following parameters: scan coverage including the whole hindfoot and ankle, volume scan mode without table movement, gantry rotation time of 400 ms, detector collimation of 64 × 0.625 mm, tube voltage of 100 kV, and tube current of 100 mA. The dynamic volume scanning started 1–3 s after intravenous bolus injection of 3 mL of iopamidol (Pamiray, 370 mg of iodine per milliliter; Dongkook Pharmaceutical, Seoul, South Korea) contrast material. Contrast material injection was followed by injection of an equal volume of normal saline, both injected with an automated dual-rail injector (Stellant; Medrad, Warrendale, Pa) at a rate of 1 mL/sec via the left auricular vein. After 5 min of rest, a disposable neonatal blood pressure cuff (Welch Allyn, NY, USA) was applied to animal’s left thigh and cuff was inflated to 200 mmHg to block blood flow to left foot for 3 min. As the cuff deflated to 0 mmHg within 3 s, a second perfusion CT was obtained in the same manner as the previous scan^23,24^. Perfusion CT protocol timeline was illustrated in Fig. 1.

### Image reconstruction and analysis of perfusion CT findings

All the CT images were transferred to an image archiving and communications workstation (INFINITT; Infinitt healthcare, Seoul, Republic of Korea) and analyzed by in-house developed perfusion analysis software^17^. The image reconstruction method for the perfusion CT data was identical to that of a prior animal experiment^17^, except that the plantar dermis in the heel base rather than that of the toes was used as the representative tissue to set the upslope time range. Each session of perfusion CT data was sent to the software and a pixel-based, color-coded perfusion map was generated by using the upslope method^17^. Based on a perfusion map, region of interest (ROI) for posterior tibial artery and plantar dermis was placed, and time-attenuation curve^1^ was calculated respectively (Fig. 1). Upslope time range was set from the arterial time-attenuation curve and the following perfusion parameters were obtained; Peak arterial enhancement (PAE), Time to peak enhancement (TTP), and maximal upslope (m). Time attenuation curve of each pixel was normalized by the maximal arterial enhancement value. Quantitative analysis of perfusion parameters was performed by two radiologists (S.H and H.D.M) in consensus. Regional blood flow at baseline perfusion CT scan was measured at the dermis of the foot sole by placing an ROI on the axial image. In the same way, ROI was set in the corresponding location of the plantar dermis on the perfusion map obtained from the second CT scan, and each perfusion value and the ratio before and after inducing CRH was calculated.

### Pathological analysis

All animals were sacrificed post CT scans and foot tissue was pathologically examined for differences in endothelial cell density. All animals were preanesthesized and sacrificed with an intravenous injection of xylazine hydrochloride. Dermal tissues of left heel of all rabbits were harvested. Tissues obtained were weighed and fixed in 10% buffered formalin and were embedded in paraffin. Thereafter, tissue samples were treated with hematoxylin and eosin staining for basic histopathological examinations and consecutive section was treated with anti-CD31 antibody (DAKO Corp., Carpinateria, CA, USA) for microvascular density (MVD) evaluation. After digital images of the histologic slides were obtained (Leica Microsystems, Mannheim, Germany), MVD was calculated using image analysis software (Image J, version 1.45 s; National Institutes of Health, Bethesda, MD)^25^. The five hot spots with the most intense vascularization were selected by J.H.L who was blinded to information of animal group, while screening at a low-power field (× 40). MVD counts of the five areas per the hot spots were performed at a high-power field (S.B.C) (× 100). Any brown-stained endothelial cells or endothelial cell clusters clearly separated from adjacent microvessels were counted as one microvessel stained by the anti-CD31 antibody, irrespective of the presence of a vessel lumen. The mean microvessel area percentage of all the measured areas was determined to be the MVD.

### Statistical analysis

All data in the study was reported as mean ± SD. The perfusion value and ratio of regional blood flow between baseline and CRH in each group was tested by using paired-T test and Wilcoxon signed rank test. The value of change of each parameter between two groups were compared using repeated measures analysis of variance test. The data processing and analysis were performed using Statistical Package for the Social Sciences version 18.0 (SPSS Inc, IBM Company, Chicago, IL). A two-sided *P* value of less than 0.05 indicates that the groups differ significantly in terms of statistical results.

## Results

### Angiography and foot perfusion CT

**Figure 2:**
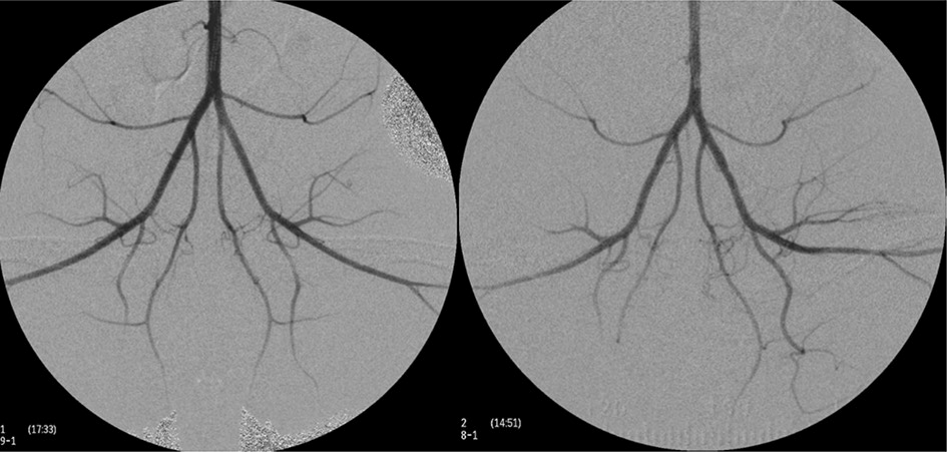
Angiography of chow-fed rabbits (left) and cholesterol-fed rabbits (right). Compared to chow-fed rabbits, mild luminal irregularity in the aortoiliac arteries were observed in cholesterol-fed rabbits.

Angiography was performed successfully in all rabbits, and there were no significant stenosis or occlusions in the aortoiliac and femorotibial arteries in both groups (Fig. 2). Foot perfusion CT was successfully performed in all rabbits from both groups at baseline and after cuff-induced reactive hyperemia (CRH) state.

### Quantitative analysis of arterial perfusion parameters at baseline and cuff-induced reactive hyperemia

**Figure 3:**
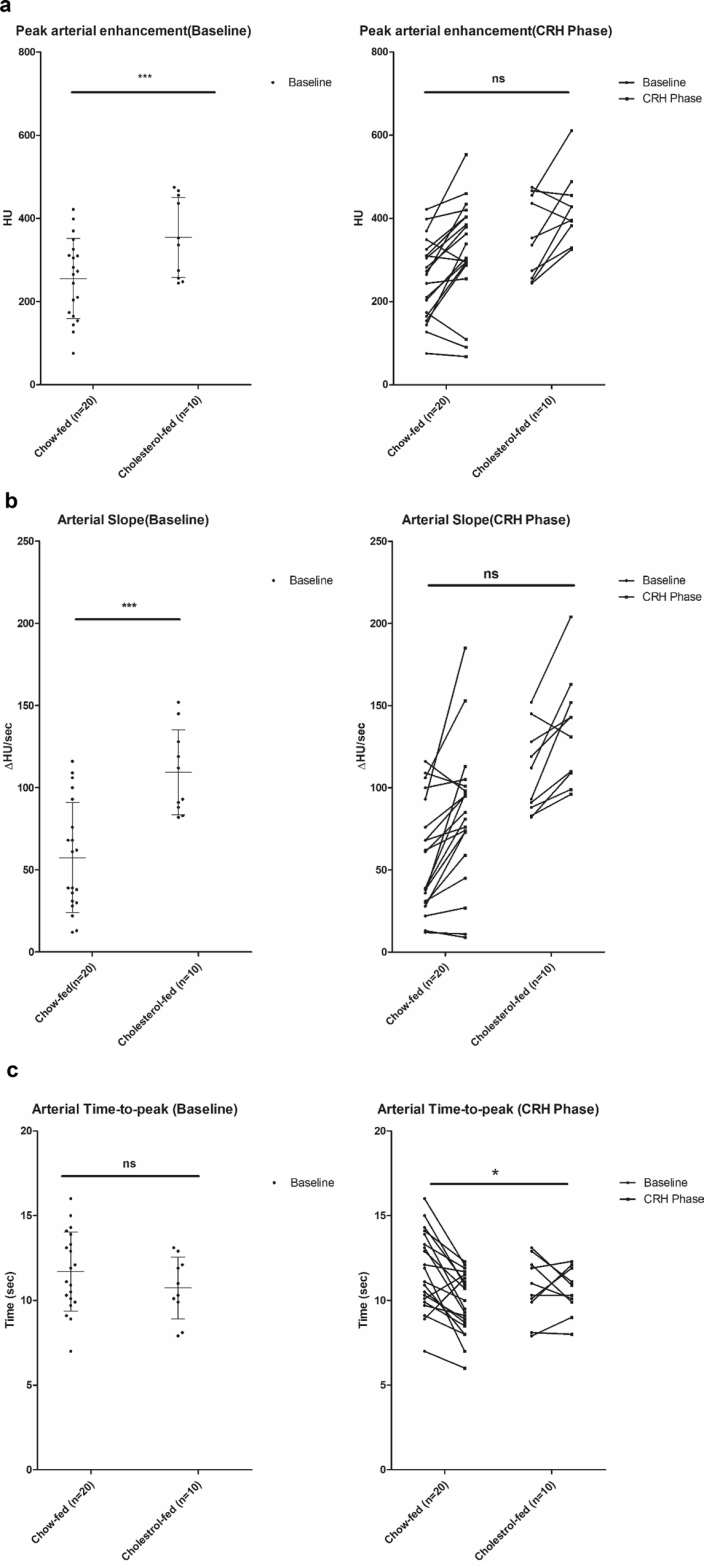
(**a**) Peak arterial enhancement. At baseline, cholesterol-fed rabbits showed increased peak arterial enhancement (PAE) compared to chow-fed rabbits (354.6 ± 96.47 HU vs 255.51 ± 96.22 HU, *p* < 0.001). During the CRH phase, both groups showed an c in PAE but intergroup differences were not significant. (**b**) Change of arterial slope. Baseline arterial velocity was higher in cholesterol rabbits compared to chow-fed rabbits (109.30 ± 25 vs 57.35 ± 33.44 HU, *p* < 0.001). During the CRH phase, there was an increasing tendency of the slope in both groups without a significant intergroup difference (*p* = 0.977). (**c**) Change of arterial time to peak. Cholesterol-fed rabbits showed no significant arterial time-to-peak (TTP) differences at baseline compared to chow-fed rabbits (10.7 ± 1.82 HU vs 11.65 ± 2.32 HU, *p* = 0.269). However, arterial TTP was accelerated (decrease in time to arterial peak) in chow-fed rabbits while no such reaction was observed in the cholesterol-fed group (*p* = 0.025). PAE = Arterial enhancement at CRH—Arterial enhancement at baseline; Hounsfield Unit (HU).

Serial measurements of arterial perfusion parameters were taken at baseline and CRH phase in both the chow-fed and the cholesterol-fed groups. At baseline, cholesterol-fed rabbits compared to chow-fed rabbits showed increased PAE, increased maximum arterial upslope, *m* but no differences in TTP (cholesterol-fed vs chow-fed rabbits; 354.6 ± 96.47 HU vs 255.51 ± 96.22 HU, *P* < 0.001; 109.30 ± 25 vs 57.35 ± 33.44 HU, *P* < 0.001; 10.7 ± 1.82 HU vs 11.65 ± 2.32 HU, *P* = 0.269, respectively) (Fig. 3). During CRH phase, both groups showed an increase in PAE and *m* compared to baseline, but intergroup differences were not significant (P = 0.922 and P = 0.977, respectively) (Fig. 3). However, the arterial TTP of the chow-fed group showed significant acceleration (decrease in time to arterial peak), whereas that of cholesterol-fed rabbits failed to show a surge (11.65 ± 2.32 s to 9.80 ± 1.76 s for chow-fed rabbits, 10.70 ± 1.82 to 10.50 ± 1.35 for cholesterol-fed rabbits, P = 0.025) (Fig. 3).

### Dermal perfusion value and perfusion ratio analysis between baseline and cuff-induced reactive hyperemia

**Figure 4:**
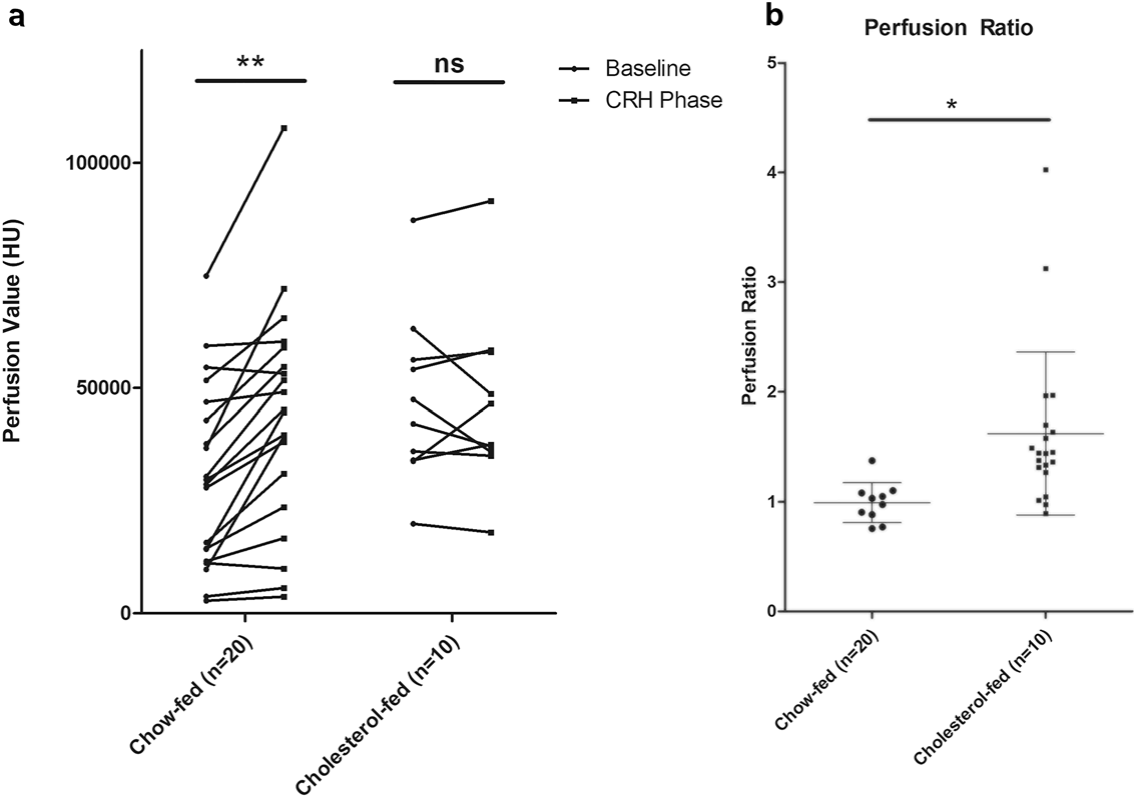
Change of foot perfusion. (**a**) Perfusion value increased in the chow-fed rabbits on CRH phase (35,040.61 ± 17,750.58 to 57,582.43 ± 22,585.11 HU; *p* = 0.008) while no such surge was demonstrated in the cholesterol-fed group (47,427.59 ± 18,968.08 to 46,692.38 ± 19,797.93 HU; *p* = 0.878). (**b**) Perfusion ratio, the ratio of perfusion value during CRH to perfusion value at baseline, showed significant drop in the cholesterol group compared to control group (*p* = 0.014).

Figure 4 summarizes the result of perfusion values of left plantar dermis in the two groups at baseline and CRH phase. Perfusion values were calculated as PAE multiplied by heal-base perfusion. There was no significant difference of dermal perfusion value between chow-fed group and cholesterol-fed group at baseline (35,040.61 ± 17,750.58 vs. 47,427.59 ± 18,968.08; *P* = 0.57) (Fig. 4a). Dermal perfusion value of chow-fed rabbits significantly increased on CRH phase (35,040.61 ± 17,750.58 to 57,582.43 ± 22,585.11 HU; *P* = 0.008), whereas no such surge was demonstrated in cholesterol-fed groups (47,427.59 ± 18,968.08 to 46,692.38 ± 19,797.93 HU; *P* = 0.878) (Fig. 4a). The perfusion ratio (dividing perfusion values of CRH to that of baseline) of left foot for normal rabbits were significantly higher than that of cholesterol-fed rabbits (1.62 ± 0.74 vs. 0.99 ± 0.18; *P* = 0.014) (Fig. 4b).

### Endothelial cell density between chow-fed and cholesterol-fed groups

**Figure 5:**
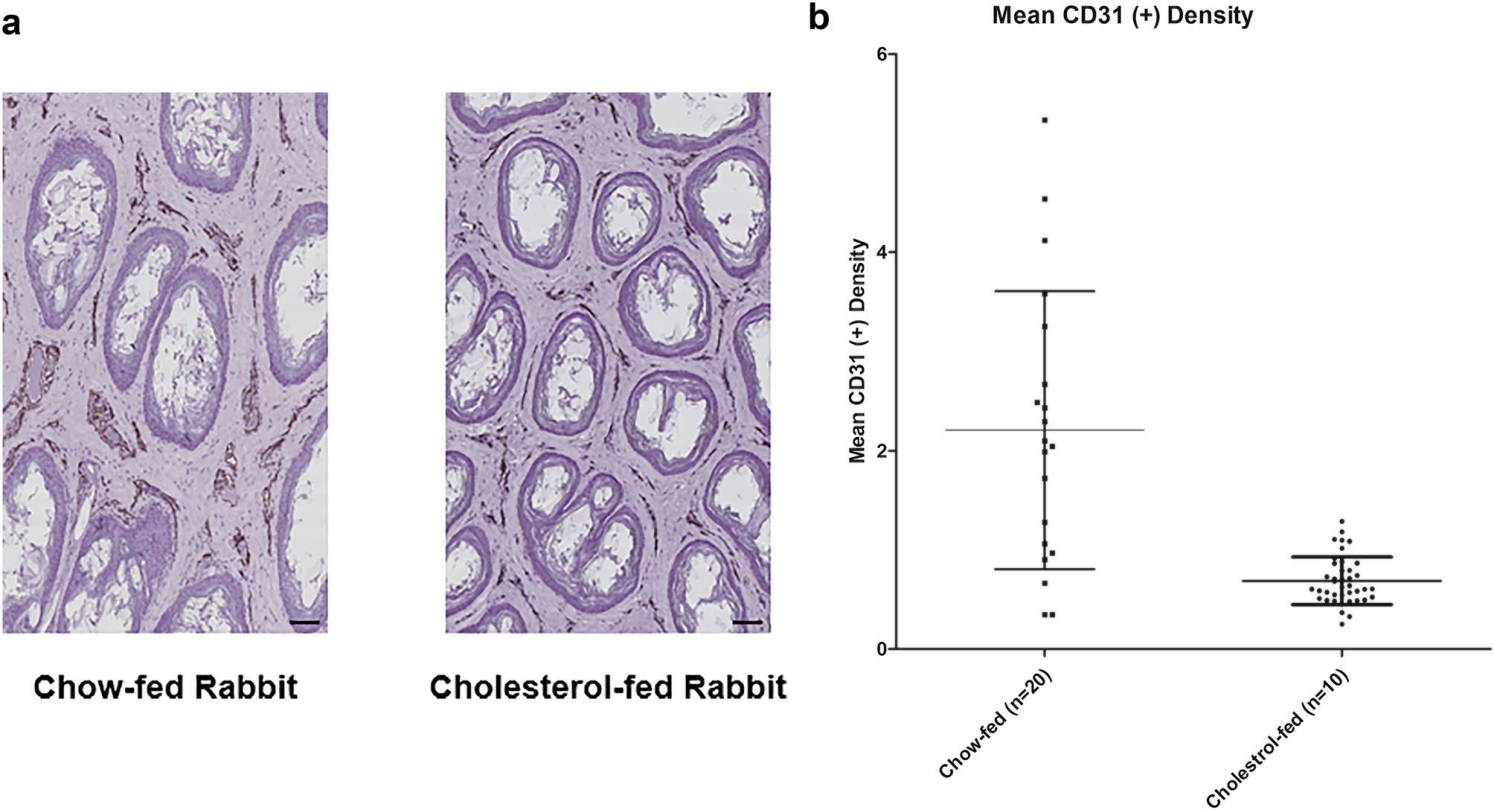
(**a**) Histologic slides of cholesterol-fed rabbits showed decreased endothelial cell density on anti-CD31 antibody staining compared to that chow-fed rabbits. Scale bars, 100 μm. (Slide images at × 100, Leica Microsystems). (**b**) Endothelial cell density as measured by anti-CD31 antibody staining showed a lower density in the cholesterol-fed rabbits compared to chow-fed rabbits (*p* < 0.01).

Endothelial cell density as measured by anti-CD 31 antibody staining showed decreased endothelial density in cholesterol fed rabbits (2.21 ± 0.31 vs. 0.69 ± 0.05; *P* < 0.01) (Fig. 5.).

## Discussion

The present study showed that the combination of dynamic volume perfusion foot CT with the upslope method and cuff-induced reactive hyperemia (CRH) was a feasible method for evaluating macrovascular reactivity and microvascular perfusion in cholesterol-fed rabbits. Using this combination, impairment of macrovascular reactivity and microvascular perfusion of the cholesterol-fed rabbits without significant macrovascular lesions were non-invasively and quantitatively demonstrated.

In this study, cholesterol-fed rabbits compared to chow-fed rabbits showed delayed TTP during CRH phase. A TTP delay on reactive hyperemia state can be explained by loss of appropriate reactivity to hyperemia at target artery due to endovascular dysfunction, leading to hypoperfusion. As atherosclerotic burden progresses, arterial TTP decreases reflecting disease severity^26–29^. Our previous study showed that the symptom of PAD patient with decreased arterial TTP was improved after revascularization therapy^17^. In contrast, our current study showed that even in the absence of macrovascular lesions, TTP is delayed on reactive hyperemia state.

Perfusion value (peak arterial enhancement multiplied by heal based perfusion), a surrogate measure of autoregulation, increased during CRH phase compared to baseline for chow-fed rabbits. However, this autoregulation did not occur in the cholesterol-fed rabbits. We hypothesized that the lack of autoregulation without any significant stenosis or occlusions in the atherosclerotic rabbits was due to endothelium dysfunction. Previous studies show that endothelium dysfunction occurs even before any apparent structural changes to vessel walls are detected on angiography or ultrasound^30^. Studies with acetylcholine test or measurements of FMD detected endothelial dysfunction at both conduit and microvascular levels in patients with coronary risk factors but no structural evidence on angiography or ultrasound^31,32^. Our study demonstrated that cholesterol-fed rabbits had a lower endothelial cell density, suggesting possible endothelium dysfunction as mechanism for impaired autoregulation during CRH phase.

Foot perfusion ratio decreased in the atherosclerotic rabbit compared to those of chow-fed rabbits. As perfusion ratio reflects surrounding microvasculature’s ability to meet tissue demands upon hyperemia, combined dynamic foot perfusion CT with CRH can be used to assess ‘vulnerable areas’ to ischemic injury. In cardiac MRI adenosine stress test, perfusion ratio, more often referred to as ‘myocardial perfusion reserve’, is already utilized to evaluate the extent of ischemic injury in myocardial infarctions^16^.

There are few studies evaluating foot perfusion using CT imaging, but these studies target patients with overt macrovascular dysfunction such as critical limb ischemia^10,33^. The present study shows the feasibility of dynamic volume perfusion foot CT to detect early microvascular dysfunction in cholesterol-fed model without macrovascular stenosis or occlusion, non-invasively and quantitatively. This is particularly important considering that currently available tools fail to provide information regarding anatomical area of decreased microperfusion, and monitor changes of microcirculation after treatment.

This study has several limitations. The cholesterol-fed rabbits may have inherent differences in pathophysiology to human atherosclerosis as atherosclerosis is a multifactorial disease developed over a long time period with species-to-species variation. In addition, rabbit models used in this study did not have significant macrovascular lesions in the large arteries which is found in human patients with peripheral artery diseases. We believed that macrovascular lesions causing significant stenosis would dictate the perfusion parameters to our study adding another variable. Also, this experiment’s goal was to see if we could detect perfusion differences in patients without significant stenosis, modeling at-risk patients without significant stenosis or PAD patients with smaller vascular lesions or PAD patients who have received intravascular therapy such as stents to alleviate macrovascular lesions. We hypothesized that if we could detect differences without macrovascular lesions, detecting perfusion differences on macrovascular lesions would be possible. However, due to this selection, we may have been unable to accurately model human patients with macrovascular lesions. The lack of macrovascular lesions in the cholesterol-fed rabbtis allowed us to measure and compare TTP and perfusion parameters in the dermis, identifying possible early changes of atherosclerosis.

Another limitation to this study is the lack of serial serum cholesterol measurements. Blood tests were performed in a limited number (n = 5) of cholesterol-fed rabbits just before angiography (9 weeks), and found serum cholesterol level of all individuals were over 400 mg/dl (data not shown). Previous study by Ribichini et al. showed that serum cholesterol levels showed no differences in chow-fed rabbits and cholesterol-fed rabbits at week 9 and week 15^34^. Yet, serum cholesterol measurements would have further clarified that microvascular perfusion impairment was not due to hypercholesterolemia.

This study was also limited to measuring one areas of foot perfusion due to the small size of the rabbit feet and a single dominant artery in the rabbit. The simpler arterial system of the rabbit’s lower extremities reduced additional variables when measuring arterial perfusion parameters. However in humans, multiple dominant arteries supply the foot and development of collateral vessels will complicate the use of foot perfusion CT.

In conclusion, macrovascular reactivity and microvascular perfusion can be measured in a cholesterol-fed rabbits by combination of perfusion foot CT using the upslope method and CRH. TTP in the tibial artery was prolonged during cuff-induced reactive hyperemic state in the cholesterol-fed rabbits compared to chow-fed rabbits suggesting delayed vasoreactivity. Microvascular perfusion parameters such as perfusion value and perfusion ratio showed impairment in the cholesterol-fed rabbits without significant macrovascular lesions, suggesting ways to detect at-risk tissues and early manifestation of atherosclerosis. Further human studies are warranted to clinically validate the usefulness of foot perfusion CT with CRH.

## Supplementary Information

Supplementary Information.

## Data Availability

All data generated or analyzed during this study are included in this published article (and its Supplementary Information files).

## Abbreviations

CRH: Cuff-induced reactive hyperemia
PAE: Peak arterial enhancement
TTP: Time to peak
